# Genome Analysis of *Alternaria alstroemeriae* L6 Associated with Black Spot of Strawberry: Secondary Metabolite Biosynthesis and Virulence

**DOI:** 10.3390/jof11100710

**Published:** 2025-09-30

**Authors:** Li Zhang, Boyuan Zhang, Lizhu Shao, Miaomiao Yang, Xueling Zhao, Ziyu Wang, Yingjun Zhang, Yuting Li, Yating Wang, Yuansen Hu, Peng Li

**Affiliations:** 1 National Engineering Research Center of Wheat and Corn Further Processing, Henan University of Technology, Zhengzhou 450001, China; zhanglibio@haut.edu.cn (L.Z.); zhang_by2021@163.com (B.Z.); slz042012@163.com (L.S.); 18436901686@163.com (M.Y.); 18837331527@163.com (Y.W.); hys308@126.com (Y.H.); 2 Institute for Complexity Science, Henan University of Technology, Zhengzhou 450001, China; 3 College of Biological Engineering, Henan University of Technology, Zhengzhou 450001, China; zyj34800@163.com (Y.Z.); lyt18692@163.com (Y.L.); 4 College of Food Science and Engineering, Henan University of Technology, Zhengzhou 450001, China; 5 School of International Education, Henan University of Technology, Zhengzhou 450001, China; 241170400430@stu.haut.edu.cn (X.Z.); 241170400526@stu.haut.edu.cn (Z.W.)

**Keywords:** *Alternaria*, antiSMASH analysis, fungi, genome sequencing

## Abstract

A pathogenic fungus was isolated from the leaves of strawberry black spot in Zhengzhou China. Based on morphological and phylogenetic analysis, the isolate was identified as *Alternaria alstroemeriae*. Hybrid sequencing and assembly yielded a high-quality 38.7 Mb genome with 12,781 predicted genes and 99.6% Benchmarking Universal Single-Copy Orthologs (BUSCO) completeness. Functional annotation revealed enrichment in carbohydrate metabolism, secondary metabolite biosynthesis, and virulence-associated genes. Strain L6 harbored 45 biosynthetic gene clusters(BGCs), including 12 clusters for terpenes, 7 for non-ribosomal peptide synthetases, and 7 for polyketide synthases. Six BGCs showed high similarity to known pathways producing alternariol (phytotoxic/mycotoxic compound), alternapyrone (phytotoxin), choline (osmoprotectant), terpestacin (anti-angiogenic agent), clavaric acid (anticancer terpenoid), and betaenone derivatives (phytotoxins). CAZyme analysis identified 596 carbohydrate-active enzymes, aligning with L6’s biotrophic lifestyle. Additionally, 996 secreted proteins were predicted, of which five candidate effectors contained the conserved RxLx [EDQ] host-targeting motif, suggesting potential roles in virulence. This genome resource highlights L6’s exceptional secondary metabolites (SMs) diversity, featuring both plant-pathogenic toxins and pharmacologically valuable compounds, indicating that this endophytic fungus is a potential producer of metabolites meriting further exploration and development.

## 1. Introduction

*Alternaria*, a genus of ascomycete fungi, is recorded with 788 species in the Species Fungorum database (https://www.speciesfungorum.org, accessed on 24 April 2025). However, due to the unresolved taxonomy of the genus, only 360 species are formally recognized [1,2]. It is a ubiquitous group growing in diverse ecosystems worldwide, found in both natural and anthropogenic environments. As facultative plant pathogens, more than 95% of *Alternaria* species infect approximately 400 plant types. The spectrum of diseases they cause, such as root and stem rot, blight, and wilt, frequently targets economically significant crops [3]. Black point is a fungal disease of strawberry, mainly associated with mycotoxigenic *Alternaria* species, which can also cause strawberry spoilage with consequent mycotoxin accumulation [4]. The diversity of the secondary metabolites (SMs) reflects *Alternaria*’s wide host range. Some SMs have received attention for their beneficial antimicrobial and pharmaceutical properties, and others for their concern as emerging phytotoxins and cytotoxins. Harmful *Alternaria* metabolites in food and food products are becoming an increasing environmental concern, but there are no regulations in place to monitor or control them [5]. Information about the genome of *Alternaria* species causing strawberry black spot is crucial to better understand the different species characteristics and for designing effective management strategies to control this disease.

Extensive studies into *Alternaria* in recent years have focused on understanding the factors that affect a wide range of SMs biosynthesis and virulence formation. *Alternaria* strains exhibit ubiquitous presence across plant ecosystems and demonstrate remarkable biosynthetic capacity with over 500 distinct SMs chemically characterized to date, as documented in the Dictionary of Natural Products database (DNP, https://dnp.chemnetbase.com; accessed on 24 April 2025). The SM repertoire of *Alternaria* encompasses diverse chemical classes, including terpenoids, pyranones, cyclic peptides, nitrogenous compounds, and hybrid derivatives, with broad-spectrum bioactivities such as antimicrobial, phytotoxic, and cytotoxic properties [6,7]. Biosynthesis of these compounds involves conserved enzymatic machineries, including modular polyketide synthases (PKSs), non-ribosomal peptide synthetases (NRPSs), hybrid NRPS-PKS systems, terpene cyclases, and ribosomally synthesized/post-translationally modified peptides (RiPPs) [8,9]. Owing to this biosynthetic complexity, genomic characterization of SMs production capacities in *Alternaria* species remains underexplored, even though their SMs represent a promising base structure for developing pesticides and drugs [10].

It is interesting that a subset of these SMs exhibit toxicological potential under specific environmental conditions, collectively classified as *Alternaria* toxins [11]. To date, over 70 structurally distinct mycotoxins with diverse biological activities have been characterized from *Alternaria* species [12]. These phytotoxic compounds are categorized into two functional classes: (1) host-selective toxins (HSTs), which specifically disrupt physiological processes in susceptible plant hosts; (2) non-host-selective toxins (NHSTs), exerting broad-spectrum cytotoxic effects across plant taxa regardless of host–pathogen compatibility [13]. Mechanistic studies show HSTs to be critical virulence determinants required for pathogen invasion and disease establishment, whereas NHSTs contribute to necrotic symptom development and ecological persistence through non-specific tissue damage [14,15].

During our investigation of fungal pathogens associated with leaf spot disease, an *Alternaria* strain L6 was isolated from symptomatic leaf tissues of *Fragaria × ananassa.* To better understand this fungus and explore its SMs biosynthetic potential and virulence, we conducted whole-genome sequencing and assembly using a hybrid approach combining next-generation sequencing (NGS) and using the Illumina HiSeq platform. Gene annotation was extensively predicted using various BLAST databases, including non-redundant (Nr) protein sequences, Swiss-Prot, Gene Ontology (GO), Kyoto Encyclopedia of Genes and Genomes (KEGG), EuKaryotic Orthologous Groups (KOG), and Carbohydrate-Active Enzyme (CAZy) databases. Additionally, the antibiotic and secondary metabolite analysis database tool (antiSMASH) was used to predict the SMs biosynthetic potential and virulence of strain L6.

## 2. Materials and Methods

### 2.1. Strain Isolation and Cultivation

Strawberry plants of 8 surveyed fields in Zhengzhou (34°828′ N, 113°545′ E), China, showed 20–30% leaf spot incidence. Leaves displayed many diseased spots ranging in diameter from 0.5 to 2.0 cm, with yellowish-brown discoloration and a noticeable reduction in the thickness of the central leaf area. Symptomatic leaves from six different strawberry plants across three fields were cut into 0.5 × 0.5 cm pieces, surface-sterilized by immersion in 2% sodium hypochlorite for 2 min, followed by 75% ethanol for 30 s, and plated on potato dextrose agar (PDA) in a 28 °C incubator.

To confirm the pathogenicity of the isolate in accordance with Koch’s postulates, spore suspensions (1 × 10^5^ spores/mL) were sprayed on healthy leaves of strawberry plants (three replicate pots in a group repeated three times), and sterile distilled water was sprayed on control plants. Plants inoculated with strawberry plants showed leaf spots, while control plants remained symptomless under incubation at 28 °C and 95% relative humidity.

### 2.2. Phylogenetic Analysis

Strain L6 was cultured on PDA at 25 °C for 5 d prior to morphological characterization. Genomic DNA was extracted to use the nuclear ribosomal internal transcribed spacer (ITS) amplification and sequencing. The ITS region was amplified by polymerase chain reaction (PCR) with ITS1/ITS4 primer pairs (5′-TCCGTAGGTGAACCTGCGG-3′/5′-TCCTCCGCTTATTGATATGC-3′) under the following conditions: 95 °C for 3 min; 30 cycles of 95 °C for 15 s, 55 °C for 15 s, 72 °C for 30 s; final extension at 72 °C for 5 min. Amplicons were verified by 1% agarose gel electrophoresis, purified using the Magen HiPure Gel Pure DNA Mini Kit (Magen Biotechnology Co., Ltd., Guangzhou, China), and commercially sequenced by Tsingke Biotechnology Co., Ltd. (Beijing, China) using Sanger methodology. The ITS sequence was subjected to Neighbor-Joining (NJ) phylogenetic analysis in MEGA X [16] with 1000 bootstrap replicates, incorporating reference sequences from typical strains in the NCBI database.

### 2.3. Genome Sequencing and Assembly Methodology

Strain L6 was cultured on PDA at 28 °C for 5 d. Genomic DNA was extracted using the SDS extraction method, and integrity and purity were evaluated by 1% agarose gel electrophoresis. Upon passing quality control, a whole-genome shotgun (WGS) strategy was adopted to construct libraries with different insert sizes [17]. Using the second-generation sequencing technology (Next-Generation Sequencing, NGS) based on the Illumina HiSeq sequencing platform, which resulted in paired-end raw data in FASTQ format, followed by useful quality control and data-filtering features using Fastp (version 0.23.4, https://github.com/OpenGene/fastp, accessed on 30 June 2025). The Unicycler (version 0.4.8) software was used to assemble and construct the scaffold sequence; meanwhile, the SPAdes (version 3.11.1, http://cab.spbu.ru/software/spades/, accessed on 30 June 2025) comprehensively evaluates the assembly results of multiple Kmers and automatically selects the optimal assembly result. Finally, an evaluation of the integrity of the predicted genomes was conducted using pleosporales_odb10 (Pleosporales database) and BUSCO (Benchmarking Universal Single-Copy Orthologs, version 2.0) [18].

### 2.4. Gene Prediction and Annotation

Gene prediction was conducted through a multi-step computational approach. Ab initio gene modeling was performed using Augustus (version 3.03), GlimmerHMM (version 3.0.1), and SNAP (2006-07-28), generating preliminary gene structure annotations [19,20,21]. Homology-based prediction was implemented using Exonerate (version 2.2.0; http://ftp.ebi.ac.uk/pub/software/vertebrategenomics/exonerate, accessed on 30 June 2025). These predictions were then integrated using EVidenceModeler (2012-07-25) to produce consensus protein-coding gene predictions through evidence-weighted reconciliation [22]. tRNA genes were predicted using tRNAscan-SE (version 1.3.1, https://github.com/UCSC-LoweLab/tRNAscan-SE, accessed on 30 June 2025), and rRNA genes were predicted using Barrnap 0.7 [23]. Long terminal repeat (LTR) sequences and transposable elements were annotated using the Extensive de novo TE Annotator (EDTA, version 6.9.5). Functional annotation of protein-coding genes was performed, including prediction of gene motifs, domains, protein functions, and metabolic pathways. For the functional annotation of putative genes, the following bioinformatics tools were applied: BLAST (version 2.10.1) for annotation against the Nr, Swiss-Prot, and KEGG databases with E-values ≤ 10^−5^.

### 2.5. Prediction of Carbohydrate-Active enZymes (CAZymes)

Carbohydrates contain significant biological information, making them valuable for analyzing the metabolic process of strain L6 and the differences between strains. The Hmmscan online annotation software (dbCAN3, https://bcb.unl.edu/dbCAN2/blast.php, accessed on 30 June 2025) was used to predict and annotate carbohydrate-active enzyme (CAZyme)-related genes in strain L6. Additionally, data from 12 other typical *Alternaria* strains obtained from the NCBI database were also compared with those of strain L6 to further explore the differences in their carbohydrate degradation capacities compared to typical species (Table S1).

### 2.6. Analysis of Secondary Metabolite Biosynthetic Gene Clusters

The SM biosynthetic gene clusters of strain L6 were predicted using antiSMASH (version 8.0.0, https://fungismash.secondarymetabolites.org/, accessed on 30 June 2025) and further annotated using BlastP analysis.

### 2.7. Secreted Protein Annotation and Prediction

Putative secreted proteins encoded in the genome of L6 were predicted using SignalPv4.0 (http://www.cbs.dtu.dk/services/SignalP/, accessed on 2 July 2025) and TMHMM-2.0 (http://www.cbs.dtu.dk/services/TMHMM-2.0/, accessed on 2 July 2025). Proteins containing an N-terminal signal peptide were identified with SignalP 4.1. Proteins predicted to contain transmembrane domains were subsequently excluded using TMHMM-2.0.

### 2.8. Prediction of Proteins with a RxLx [EDQ]

The predicted secreted proteins in strain L6 were screened for the conserved host-targeting motif RxLx [EDQ] using the MEME prediction server (http://meme-suite.org/tools/meme, accessed on 2 July 2025) with default parameters. Structural visualization analysis of protein-coding genes was performed using GSDS 2.0 (https://gsds.gao-lab.org/, accessed on 2 July 2025). Domain analysis and functional enrichment of domains were performed for the unknown proteins using SMART (http://smart.embl.de/, accessed on 2 July 2025) [24].

## 3. Results

### 3.1. Morphology and Pathogen Verification of Strain L6

**Figure 1:**
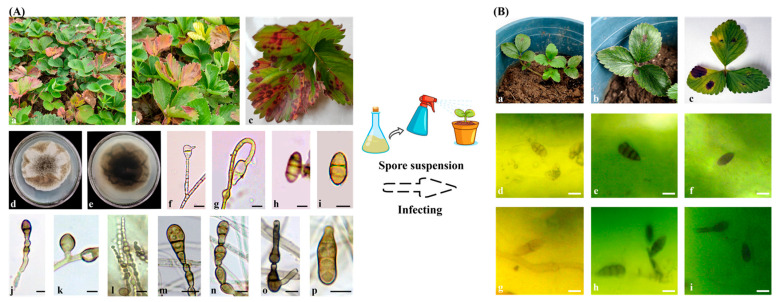
(**A**) Colony and spore morphology of *Alternaria alstroemeriae* L6. (**a**–**c**), Extensive onset on Strawberry plants; (**d**,**e**), Colonies on PDA above and below. (**f**–**p**), Conidia. Scale bars: (**f**–**p**) = 10 μm; (**B**), *A. alstroemeriae* L6 infects strawberry leaves. (**a**–**c**), The infection of strain L6 on strawberry leaves. (**d**–**i**) The spore of strain L6 in the leaves of diseased strawberries. Scale bars: (**d**–**i**) = 10 μm.

After 4–5 days of incubation at 28 °C, four morphologically similar fungal isolates were obtained and purified. ITS sequencing analysis confirmed that all four isolates belonged to *Alternaria alstroemeriae*. One representative isolate, designated L6, was selected for further study. Strain L6 developed after culturing on a PDA medium for 3–4 d that its white colonies grew rapidly on the PDA medium at the initial stage, the brown mycelia appeared at the edge of the colony after 5–6 d, and the whole surface of the mycelia was grayish brown after 10 d. Microscopically, the conidial peduncle of the strain was grayish brown, and the mycelia were compartmentalized, 2.1 to 5.0 μm (mean 3.6 μm) wide, with some hyphae exhibiting 1 to 3 branches. Conidia were 16.2–21.1 × 9.4–10.2 μm (mean 18.8 × 9.8 μm), brown, ellipsoid, with 1–3 transverse septa and 0–2 longitudinal septa, constricted at the septa and taupe in color (Figure 1A). The morphological features were consistent with those of *A. alstroemeriae* [25,26].

All strawberry plants, which were sprayed with spore suspensions of strain L6, showed disease in the leaves. After inoculating strawberry plants that exhibited leaf spot symptoms, the morphology and sequences of re-isolated fungal isolates from the tested plants were the same as the original isolate (Figure 1B).

### 3.2. Phylogenetic Analysis of Strain L6

**Figure 2:**
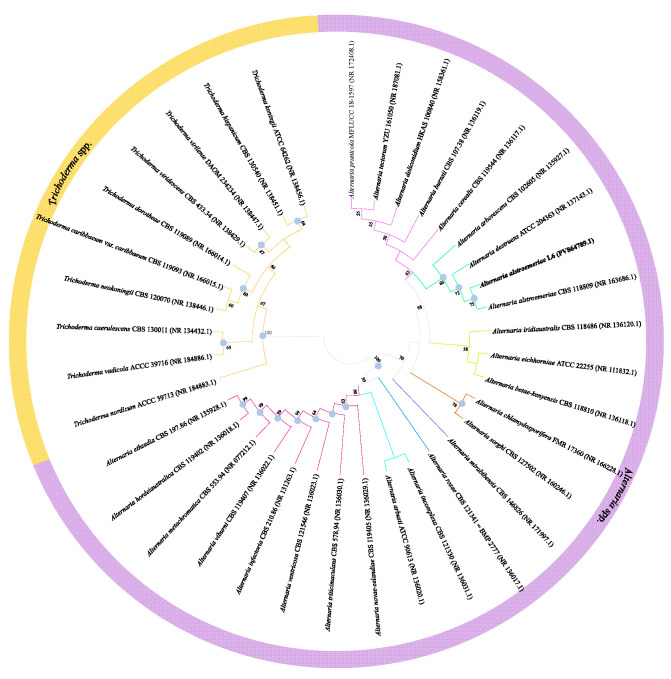
ITS-based Neighbor-Joining phylogenetic tree of strain L6. Filled circles indicate that the corresponding nodes were also recovered by the neighbour-joining and maximum likelihood algorithms. Numbers at nodes are bootstrap percentages >50% (based on 1000 resamplings).

Subsequently, a Neighbor-Joining phylogenetic tree was constructed based on the ITS sequence of strain L6 (Genbank accession: PV864789.1). The tree indicated that strain L6 shared the highest ITS sequence homology with *A. alstroemeriae* strain CBS118809 (GenBank accession: MH863036.1), with 99.80% similarity, and phylogenetically clustered within the same clade as *A. alstroemeriae* CBS118809. Combined with morphological characteristics, these results confirm that strain L6 belongs to the genus *Alternaria* and was identified as *A. alstroemeriae* (Figure 2).

### 3.3. Results of Genome Sequencing and Assembly

The whole genome sequence of strain L6 has been submitted to NCBI under the accession number JBRANC000000000. The genome assembly of L6 yielded 38.7 Mb with 50.84% GC content and an N50 value of 1,029,672 bp (Table 1). BUSCO assessment revealed 99.6% completeness, indicating high assembly quality (Table S2). The total CDS length was 17,294,764 bp, accounting for 44.00% of the genome.

Comparative genomic analysis with typical *Alternaria* strains from NCBI showed that strain L6 had a larger genome size and higher gene count (Table S1). Genomic prediction identified 222 tRNAs, 12,559 mRNAs, and 47 rRNAs (Table 2).

### 3.4. Genome Annotation

**Figure 3:**
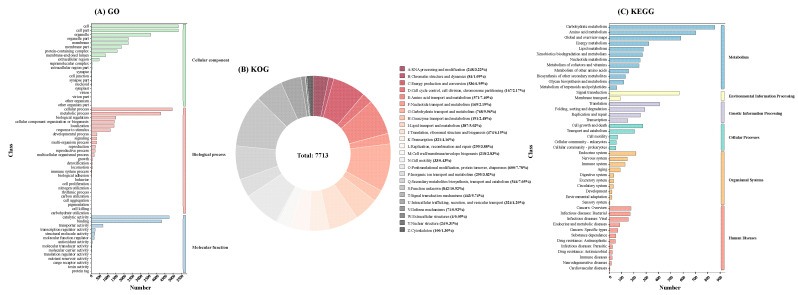
Functional annotation of strain L6 genes encoding the proteins. (**A**), Gene Ontology (GO) analysis; (**B**), euKaryotic Orthologous Group (GO); (**C**), Kyoto Encyclopedia of Genes and Genomes (KEGG) analysis.

Functional annotation of strain L6 genes was performed by aligning putative protein-coding sequences against the NR, KOG, KEGG, Swiss-Prot, and GO databases using BLAST analysis (Table S3). A total of 7555 genes were annotated into three Gene Ontology (GO) categories: cellular component, biological process, and molecular function, encompassing 60 subcategories (Figure 3A). Molecular functions were primarily distributed across catalytic activity, binding activity, and transporter activity. In biological processes, cellular processes, metabolic processes, and biological regulation accounted for the highest proportions. In contrast, molecular functions in *Alternaria* sp. SPS-2 mainly involved catalytic activity, ion binding, and oxidoreductase activity [27]. Among 24 KOG functional categories, most genes were associated with unknown functions (10.92%), followed by Carbohydrate transport and metabolism (9.96%), Post-translational modification, protein turnover, chaperones (7.78%), Amino acid transport and metabolism (7.40%), and Secondary metabolites biosynthesis, transport and catabolism (7.05%) (Figure 3B). This high proportion of uncharacterized genes highlights the strain’s potential for further genomic exploration. KEGG annotation assigned 7938 genes to 45 pathways (Figure 3C), with carbohydrate metabolism and amino acid metabolism being the most enriched. These abundant metabolic functions suggest high energy conversion efficiency.

### 3.5. CAZyme Analysis

**Figure 4:**
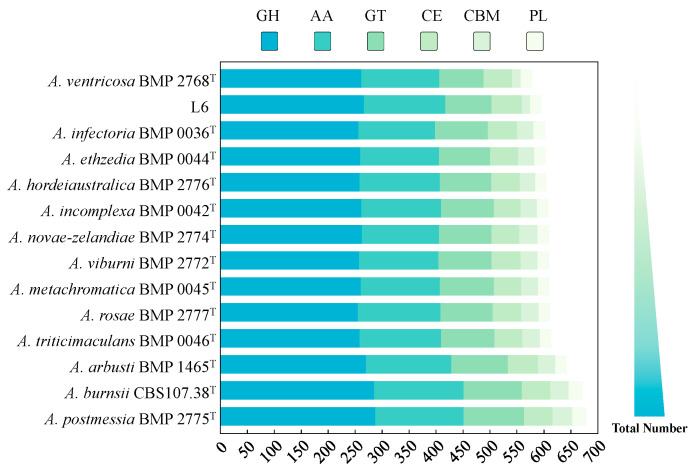
CAZyme profiles of strain L6 and other typical *Alternaria* strains deposited in CAZyme database.

Carbohydrates, also known as saccharide compounds, are the most abundant and widely distributed organic compounds in nature, serving as the primary energy source for all organisms to sustain life activities. Enzymes acting on carbohydrate complexes, oligosaccharides, and polysaccharides constitute the most structurally diverse protein assemblies on Earth. Fungi exhibit a high degree of substrate metabolic specificity adapted to their habitats by secreting diverse CAZymes. A total of 596 CAZyme family genes were annotated in strain L6, including 267 glycoside hydrolases (GHs), 151 auxiliary activities (AAs), 56 carbohydrate esterases (CEs), 86 glycosyltransferases (GTs), 21 polysaccharide lyases (PLs), and 15 carbohydrate-binding modules (CBMs) (Figure 4; Table S4). Compared to other standard strains in the *Alternaria* genus, this pathogenic fungus isolated from infected strawberry leaves harbors fewer annotated CAZyme family genes, likely due to its classification as a biotrophic fungus, which inherently possesses fewer CAZymes than other fungi [28]. Subsequent experiments on strain L6 infecting strawberry leaves confirmed its identity as a biotrophic fungus (Figure 1B).

### 3.6. Analysis of Secondary Metabolite Biosynthetic Potential

**Figure 5:**
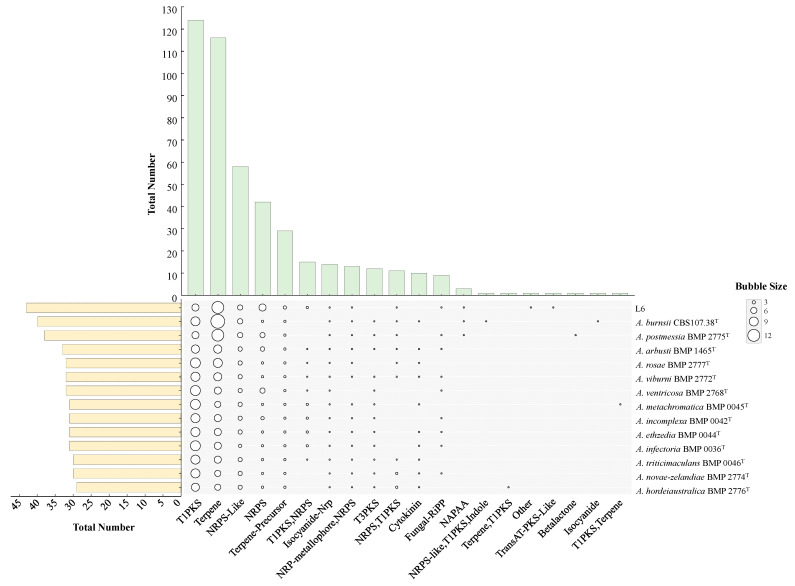
The number and type of secondary metabolite BGCs in strain L6 and other typical *Alternaria* strains are deposited in the antiSMASH database. Green indicates the number of a specific type of SM across all strains; yellow shows the number of all metabolites in a specific strain.

The antiSMASH results indicated that Type I polyketide synthases (T1PKSs) were the most abundant in the biosynthetic gene clusters (BGCs) of secondary metabolites in the annotated strain, followed by terpenes. However, compared to 12 other reference *Alternaria* strains, strain L6 harbored the highest number of BGCs (Figure 5).

**Figure 6:**
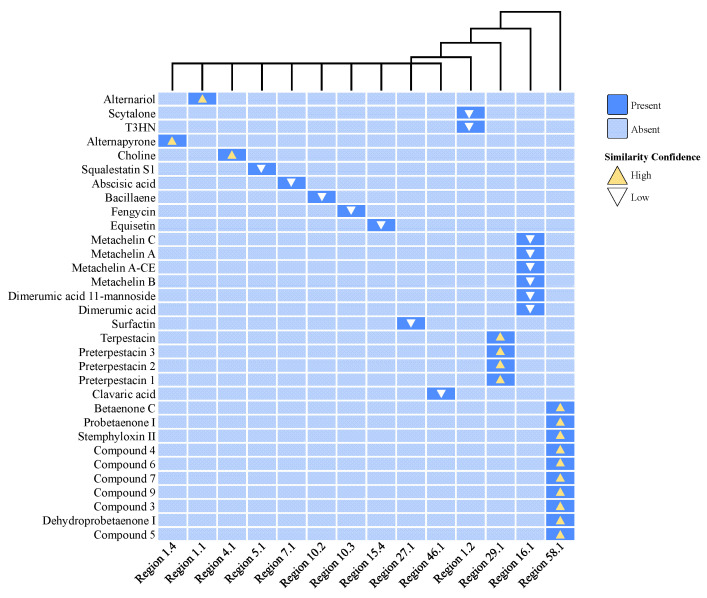
The identified secondary metabolite regions in strain L6 and their most similar known cluster are shown in a binary plot. According to the prediction information from the antiSMASH database, light blue indicates that no corresponding product was predicted (Absent), while dark blue indicates that the corresponding product was predicted (Present). The triangle symbols represent similar substances predicted for the relevant gene clusters, with yellow indicating higher similarity and white indicating lower similarity.

The BGC composition includes 12 terpenes, 7 non-ribosomal peptide synthetases (NRPSs), 7 T1PKSs, 5 NRPS-like fragments, 3 terpene precursors, 2 T1PKS/NRPSs, and one each of other types (e.g., fungal-RiPP and cytokinin). Of these, six BGCs showed high similarity to known functional gene clusters, which are putatively responsible for producing compounds such as alternariol, alternapyrone, choline, terpestacin, clavaric acid, betaenone C, and dehydroprobetaenone I (Figure 6; Table S5). However, the biosynthetic products of the remaining BGCs in strain L6 remain uncharacterized and are yet to be elucidated.

**Figure 7:**
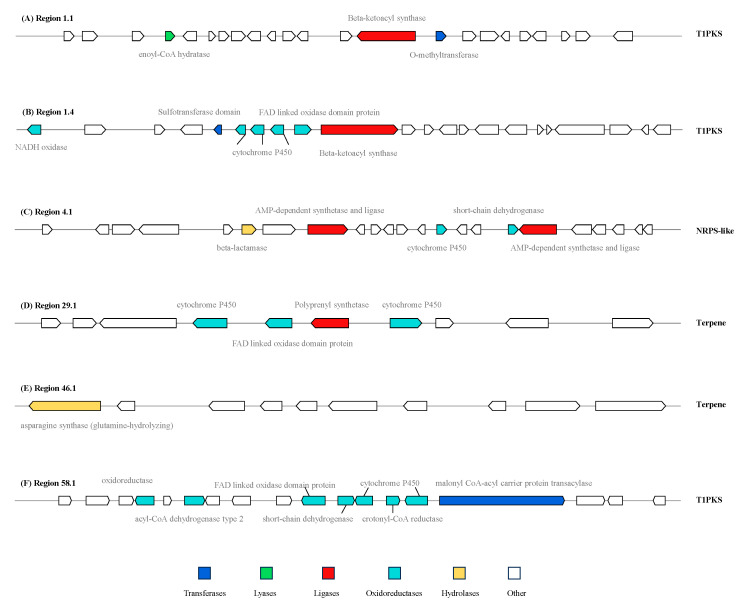
Six identified BGCs with high identity in strain L6. (**A**) Region 1.1: TIPPKS-related gene cluster with malonyl-CoA ligase, beta-ketoacyl synthase, and oxidoreductase; (**B**) Region 1.4: TIPPKS-related gene cluster including NADH oxidase, Nannochloropsis-derived P450 hydroxylase domain, and beta-ketoacyl synthase; (**C**) Region 4.1 was an NRPS-like gene cluster; (**D**) Region 32.1 was a Type I PKS gene cluster; (**E**) Region 46.1 was a Type I PKS gene cluster; (**F**) Region 58.1 was a TIPPKS-related gene cluster. Different colors represent different types of enzymes: blue for Transferases, green squares for lyases, red for ligases, turquoise for oxidoreductases, yellow for hydrolases, and white for others.

Region 1.1 has been annotated as alternariol (AOH) and displayed high similarity with the BGC of AOH from *Parastagonospora nodorum* SN15 (GenBank: KP941080.1). The core biosynthetic gene FUN_000234 is a PKS-related enzyme, showing high homology with the key AOH synthesis gene polyketide synthase *pksI* (JX103644.1). This gene typically co-expresses with O-methyltransferases, FAD-dependent monooxygenases, short-chain dehydrogenases, and other enzymes to produce alternariol derivatives. As a toxic metabolite of *Alternaria* fungi, AOH exerts its effects by disrupting glutathione metabolism, interfering with redox system-related enzymes, and inducing DNA damage [29]. Interestingly, recent studies have revealed its diverse potential pharmacological activities, including cytotoxic effects mediated by reactive oxygen species (ROS)-induced oxidative stress and mitochondrial dysfunction, anti-inflammatory properties, cell cycle arrest, apoptotic cell death, genotoxicity, mutagenicity, anti-proliferative effects, autophagy modulation, along with estrogenic and clastogenic mechanisms, making it a promising candidate for a chemotherapeutic agent (Figure 7A; Figure S1A) [30].

Region 1.4 exhibits significant similarity with the BGC of alternapyrone in *A. solani* (GenBank: AB120221.1). The *pksC* gene (JX103638.1), with 99.96% similarity, is critical for alternapyrone biosynthesis. Alternapyrone is a decaketide synthesized by a type I polyketide synthase in *A. solani*. Structurally, it is a 2H-pyran-2-one derivative in which the hydrogens at positions 3, 4, 5, and 6 are replaced by a methyl, hydroxy, methyl, and (4*E*,6*E*,12*E*)-4,6,8,12,14-pentamethylhexadeca-4,6,12-trien-2-yl group, respectively. It is classified as an olefinic compound, a 2-pyranone, a heteroaryl hydroxy compound, and a decaketide. Notably, certain compounds in this class exhibit phytotoxic effects on wheat seed germination (Figure 7B; Figure S1B) [31].

Region 4.1 belongs to a BGC of choline containing an NRPS gene (GenBank: AN5318.2), which has been confirmed to participate in the biosynthesis of choline-related secondary metabolites in *Aspergillus nidulans* [32]. These metabolites serve as essential compounds for filamentous fungal growth, play a crucial role in hyphal morphology regulation, and act as vital micronutrients critical for cellular and organismal homeostasis [33,34]. Studies have shown that the choline-glycine betaine biosynthesis pathway plays a key physiological role in bacterial adaptation to high osmotic stress (Figure 7C; Figure S1C) [35].

Region 46.1 was found to harbor clavaric acid, an anticancer terpenoid compound that also acts as a reversible farnesyltransferase inhibitor with an IC50 of 1.3 μM [36,37]. The core terpene gene in this region exhibits high sequence similarity to EU665687.1 within BGC0001248 (Figure 7E; Figure S1D).

Region 29.1 and Region 58.1 contain biosynthetic gene clusters (BGCs) capable of synthesizing diverse compounds. The BGC in Region 29.1, classified as a terpene cluster (GenBank: KB445573.1), can produce terpestacin, preterpestacin 3, preterpestacin 2, or preterpestacin 1. Terpestacin, a small fungal toxin, exhibits phytotoxic effects but also inhibits tumor angiogenesis by targeting Ubiquinol-Cytochrome C Reductase Binding (UQCRB) of mitochondrial complex III, suppressing hypoxia-induced ROS production, and disrupting cellular oxygen sensing [38]. Among the preterpestacins, preterpestacin 1 has been studied most extensively and is implicated in diverse functions, including cellular metabolism and enzymatic substrate interactions (Figure 7D; Figure S1E) [39].

The BGC in Region 58.1 corresponds to an unidentified polyketide synthase (PKS) cluster, showing similarity to the betaenone C cluster in *P. nodorum* (GenBank: CH445335.1). In addition to betaenone C, this cluster may generate probetaenone I (a biosynthetic intermediate of the phytotoxin betaenone B), stemphyloxin II (a novel phytotoxic compound), compound 3-5 (unknown structure), and dehydroprobetaenone I (a lead compound for drug discovery) (Figure 7F; Figure S1F) [40,41,42]. The discovery of these biosynthetic clusters provides a robust foundation for identifying metabolites associated with strain L6, while highlighting strain L6 as a significant source of bioactive terpenoids.

### 3.7. Metabolism of Terpenoids and Polyketides in L6

Based on the antiSMASH database results, L6 was predicted to induce blackening and decay of strawberry leaves primarily by producing phytotoxic metabolites, including terpenoids and polyketides. Five metabolic pathways related to terpenoids and polyketides were annotated in strain L6: Terpenoid backbone biosynthesis, Limonene and pinene degradation, nonribosomal peptide structures, Carotenoid biosynthesis, and Geraniol degradation (Table 3).

**Figure 8:**
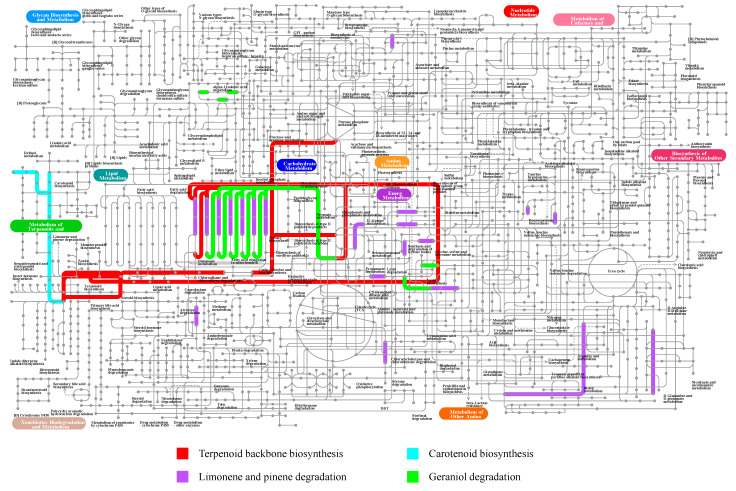
Based on the whole-genome KEGG functional annotation results of strain L6, the iPath online tool was used to visualize the five annotated metabolic pathways related to the metabolism of terpenoids and polyketides.

Based on the whole-genome KEGG functional annotation results of strain L6, the annotated metabolic pathways related to terpenoids and polyketides were visualized using the iPath online tool, with the results presented in Figure 8 (The pathway map for nonribosomal peptide structures (ko01054) is shown in Figure S2). Taking the terpenoid backbone biosynthesis pathway as an example, terpenoid synthesis in strain L6 initiates from C5 units, primarily synthesized via the mevalonate pathway. Subsequently, these C5 building blocks undergo condensation catalyzed by prenyltransferases to form longer-chain isoprenoid precursors. The synthesis of C10–C20 isoprenoid compounds occurs predominantly in the cytoplasm and relies on the mevalonate pathway.

### 3.8. Prediction of Secreted Proteins and RxLx [EDQ] Effector Candidates

Using SignalP V4.1 and TMHMM-2.0 to predict N-terminal signal peptides and transmembrane domains of ORFs, respectively, we screened the genome of strain L6 for secreted protein-coding genes that may serve as effector candidates. A total of 996 secreted protein-coding genes were identified, accounting for 7.8% of the predicted genes.

**Figure 9:**
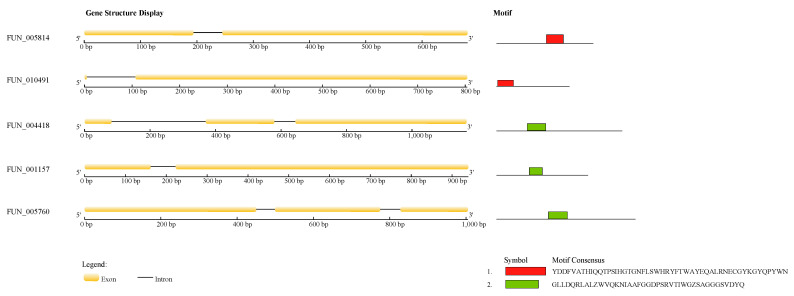
Illustration of candidate effector molecules containing 5 RxLx motifs.

“The secretome” refers to the collection of proteins containing signal peptides, which are processed via the endoplasmic reticulum and Golgi apparatus before secretion [43]. Secreted proteins are common across organisms from bacteria to humans and serve diverse functions. These functions include roles in the immune system [44], neurotransmitter activity in the nervous system [45], hormone/pheromone signaling [46], nutrient acquisition [47,48,49], cell wall construction and remodeling [50], signal transduction and environmental sensing [51], as well as competition with other organisms [52,53,54,55]. Some secreted proteins in pathogens act as effectors with specialized features to manipulate and/or disrupt host cells. In Plasmodium and Phytophthora species, effectors carry the RXLX [EDQ] or RXLR motif as host-targeting signals. Since strain L6 was pathogenic to strawberry leaves, the 996 secreted proteins identified in L6 were further analyzed, revealing 42 that contained the RxLx [EDQ] motif domain. As cloned effectors interacting with plants in fungi and oomycetes are typically no longer than 300 amino acids, additional screening identified secreted proteins possessing the RxLx [EDQ] motif within 300 amino acids downstream of the N-terminal signal peptide. This process yielded five effector candidate proteins harboring two conserved RxLx [EDQ] domains (Figure 9).

We performed homology analysis of the five effector proteins using the BLASTp (NCBI, default parameters). Results showed that FUN_010491 and FUN_004418 were annotated as hypothetical proteins, while FUN_005814, FUN_001157, and FUN_005760 were annotated as a di-copper center-containing protein, a carbohydrate esterase family 1 protein, and an uncharacterized protein J4E82_008103, respectively. Remarkably, FUN_001157 and FUN_005760 exhibited 100% sequence homology in the alignment (Table 4).

Domain and enrichment analyses revealed no significant association between the domains of these five proteins and pathogenicity. However, given the critical role of RxLR effectors in the pathogenic mechanisms of certain fungi, functional characterization of proteins harboring the RxLx [EDQ] motif in the secretome of L6 will be a key focus of subsequent research [56].

## 4. Discussion

*Alternaria* is a common cause of black spot disease, capable of both plant parasitism and saprophytism in the natural environment. As a pathogen, it infects crops like sugar beet (*Beta vulgaris*) [57] and Labiatae (*Ocimum basilicum*) [58], commonly causing leaf spot [59]. The *Alternaria* can also survive in soil and on plant residues, under suitable conditions, it spreads easily between environments and hosts. Therefore, diseases caused by this fungus require serious attention [60]. This study demonstrates that *A. alstroemeriae* can cause leaf spot disease in strawberries in Zhengzhou, China.

To further explore the pathogenic mechanism, a high-quality de novo genome of *A. alstroemeriae* L6 was obtained via whole-genome sequencing and assembly. The genome of strain L6 is 38.7 Mb, larger than most sequenced *Alternaria* strains. It has a high BGCs count (45 clusters), underscores substantial secondary metabolite (SM) potential, dominated by T1PKS, terpene, and NRPS systems. The identification of BGCs for alternariol, alternapyrone, and betaenone derivatives aligns with L6’s role as a strawberry pathogen and holds critical significance for plant pathology research and disease management.

These phytotoxins are inferred to disrupt host physiology through multiple potential mechanisms: alternariol interferes with glutathione metabolism and induces DNA damage [29], alternapyrone inhibits wheat seed germination by targeting early embryonic development [31], and betaenone derivatives cause necrotic tissue damage [40]. The presence of these toxin-encoding gene clusters not only provides molecular markers for rapid pathogen diagnosis but also identifies novel targets for disease control. For example, developing small-molecule inhibitors against the beta-ketoacyl synthase (a core enzyme in T1PKS-mediated alternapyrone synthesis) could block toxin production, reducing the severity of leaf spot without relying on broad-spectrum fungicides—alleviating environmental pressure and delaying the evolution of fungicide resistance.

Notably, the presence of biosynthetic gene clusters for compounds with reported anticancer properties—such as clavaric acid and terpestacin—highlights the potential of strain L6 as a valuable source of bioactive molecules. The identification of the terpene biosynthetic gene cluster responsible for clavaric acid production (Region 46.1) suggests promising opportunities for future biotechnological exploitation. For instance, heterologous expression of this cluster in engineered microbial hosts could facilitate improved production yields, thereby enabling more efficient exploration of its pharmacological properties. Furthermore, clavaric acid is known to irreversibly inhibit β-lactamase activity, enhancing the efficacy of penicillin and cephalosporin antibiotics against enzyme-producing resistant bacteria [61]. Such an approach may eventually provide a more stable and scalable source of clavaric acid for further preclinical drug development. Similarly, terpestacin (encoded by Region 29.1), which has been reported to inhibit tumor angiogenesis through binding to UQCRB in mitochondrial complex III [38], offers promising avenues for future research. Characterization of its gene cluster may support structure–activity relationship (SAR) studies.

The relatively low CAZyme count (596 genes) compared to other typical *Alternaria* strains, combined with the dominance of GHs and AAs, leads us to postulate that strain L6 employs targeted degradation of plant cell wall components during early invasion. Importantly, this CAZyme profile may synergize with secondary metabolite production: GH-mediated breakdown of strawberry cell wall cellulose and hemicellulose not only creates physical entry points for L6 but also releases oligosaccharide signals that can potentially induce the expression of toxin biosynthetic gene clusters [62]. To further validate this hypothesized mechanism, we plan to conduct integrated metabolomic and transcriptomic analyses to elucidate the precise pathogenic strategies employed by strain L6.

Notably, five secreted proteins harboring the RxLx [EDQ] effector motif were identified. While homology analysis linked two to hypothetical proteins and others to carbohydrate esterases or copper-binding domains, no domains were directly associated with established virulence mechanisms. This suggests L6 may employ novel effectors or that RxLx [EDQ] motifs function synergistically with other uncharacterized domains. The presence of these motifs—analogous to RxLR effectors in oomycetes-hints at conserved strategies for host manipulation, though functional validation is needed.

The *A. alstroemeriae* can cause plant disease, such as in *Nicotiana tabacum* L. and *Caryota mitis* L. [25,63]. In this experiment, we report *A. alstroemeriae* causing leaf spot on strawberry in China, its growth leads to plant wilting and death, potentially causing economic loss. Additionally, some species within the *Alternaria* genus produce toxins; future research will focus on their toxin production and control in *A. alstroemeriae*. In this study, we sequenced and analyzed the genome of strain L6 to assess its plant-pathogenic toxins and pharmacologically valuable compounds, which can provide a reference basis for diagnosing black spot disease caused by *A. alstroemeriae* on strawberry.

## Figures and Tables

**Table 1:** General genomic features of strain L6.

Item	Value
Total length	38,715,194
Max length	5,489,576
GC content	50.84%
N50	1,029,672
Counts of scaffold number	2165
Total genes length	18,851,088
Total genes number	12,781
Total CDSs length (bp)	17,294,764
CDSs percentage of genome	44.00%
Total length	38,715,194
Max length	5,489,576
GC content	50.84%
N50	1,029,672
Sequencing depth	161x

**Table 2:** Genomic assembly and functional annotation of strain L6.

Type	Number	In Genome (%)
tRNA	222	0.05
mRNA	12,559	48.64
rRNA	47	0.02
LTR Gypsy	53	0.20
LTR Unknown	45	0.12
Transposable Element	161	0.58

**Table 3:** KEGG gene annotation of terpenoids and polyketides metabolism in strain L6.

Term	ID	Gene Number
Terpenoid backbone biosynthesis	ko00900	31
Limonene and pinene degradation	ko00903	9
Nonribosomal peptide structures	ko01054	8
Carotenoid biosynthesis	ko00906	6
Geraniol degradation	ko00281	6

**Table 4:** List of predicted effector candidates carrying the RxLx [EDQ] motif.

Name	Size (aa)	Start of Sequence	Annotation
FUN_005814	209	MRFCSLPTAILALASLVESAALQPRDLLQDLQDQALAALKE	Di-copper centre-containing protein
FUN_010491	233	MSAIFASLVVVLVAVILSKKNRGSFLDDTSEHIERMD	hypothetical protein
FUN_004418	271	MFASIAILQVLCAAVASAQLTSKLTEIIPWASLGDEYGFI	hypothetical protein
FUN_001157	292	MHFSTSVLGSIVAFCATANAALTRVNDFGANPSNLQMNIYV	carbohydrate esterase family 1 protein
FUN_005760	300	MRFLTAVTSFLSVAAAATLGKRAVTPGTLSQVTSFGAAPTK	uncharacterized protein J4E82_008103

## Data Availability

The whole genome sequencing data for strain L6 have been deposited in the GenBank database under the accession number JBRANC000000000. The original contributions presented in this study are included in the article/Supplementary Material. Further inquiries can be directed to the corresponding author(s).

## Supplementary Materials

The following supporting information can be downloaded at: https://www.mdpi.com/article/10.3390/jof11100710/s1, Table S1: Genome assembly integrity assessment for strain L6; Table S2: Typical strains genomic features of *Alternaria* available in the NCBI database; Table S3: NR, KOG, KEGG, Swiss-Prot, and GO databases in strain L6; Table S4: CAZyme profiles of strain L6 and other typical *Alternaria* strains deposited in CAZyme database; Table S5: Secondary metabolite BGCs of strain L6 by antiSMASH analysis; Figure S1: Six identified BGCs with high identity in strain L6 responsible for biosynthesis; Figure S2: The pathway map for nonribosomal peptide structures (ko01054).
