# Supplementary material for: Genome Analysis of Alternaria alstroemeriae L6 Associated with Black Spot of Strawberry: Secondary Metabolite Biosynthesis and Virulence
Source: J Fungi (Basel). 2025 Sep 30;11(10):710. doi: 10.3390/jof11100710 (PMC12565302; doi:10.3390/jof11100710)
Supplement: Supplementary file 1 [file jof-11-00710-s001.zip › jof-3858490-supplementary.pdf]

Journal of Fungi

MDPI

Supplementary Materials for

Genome Analysis of *Alternaria alstroemeriae* L6 Associated with Black Spot of Strawberry: Secondary Metabolite Biosynthesis and Virulence

Li Zhang [1,2,3], Boyuan Zhang [1,4], Lizhu Shao [1,3], Miaomiao Yang [1,3], Xueling Zhao [5], Ziyu Wang [5], Yingjun Zhang [3], Yuting Li [3], Yating Wang [1,3], Yuansen Hu [1,3] and Peng Li [2,*]

1 National Engineering Research Center of Wheat and Corn Further Processing, Henan University of Technology, Zhengzhou 450001, China; zhanglibio@haut.edu.cn (L.Z.); zhang_by2021@163.com (B.Z.); slz042012@163.com (L.S.); 18436901686@163.com (M.Y.); 18837331527@163.com (Y.W.); hys308@126.com (Y.H.)
2 Institute for Complexity Science, Henan University of Technology, Zhengzhou 450001, China
3 College of Biological Engineering, Henan University of Technology, Zhengzhou 450001, China; zyj34800@163.com (Y.Z.); lyt18692@163.com (Y.L.)
4 College of Food Science and Engineering, Henan University of Technology, Zhengzhou 450001, China
5 School of International Education, Henan University of Technology, Zhengzhou 450001, China; 241170400430@stu.haut.edu.cn (X.Z.); 241170400526@stu.haut.edu.cn (Z.W.)
* Correspondence: lipeng@haut.edu.cn

Table S1. Typical strains genomic features of *Alternaria* available in the NCBI database.

Strain	Size (Mbp)	GC %	N50 (kb)	Sequencing technology	Gene	Genbank
A. burnsii CBS107.38^T	32.96	51.0	1805.72	Illumina HiSeq	11,314	GCA_013036055.1
A. incomplexa BMP 0042^T	33.46	52.5	1850.41	Illumina HiSeq	11,090	GCA_024043165.1
A. triticimaculans BMP 0046^T	36.79	50.0	1666.52	Illumina HiSeq	11,086	GCA_023758025.1
A. arbusti BMP 1465^T	33.98	52.5	1678.45	Illumina HiSeq	11,826	GCA_024043155.1
A. ventricosa BMP 2768^T	34.69	51.5	1329.36	Illumina HiSeq	11,061	GCA_023758065.1
A. novae-zelandiae BMP 2774^T	37.57	49.5	1242.97	Illumina HiSeq	11,027	GCA_023758075.1
A. infectoria BMP 0036^T	36.52	50.0	1572.73	Illumina HiSeq	11,092	GCA_024043175.1
A. viburni BMP 2772^T	36.35	50.5	1053.82	Illumina HiSeq	11,856	GCA_023758015.1
A. metachromatica BMP 0045^T	37.28	50.0	1012.32	Illumina HiSeq	11,107	GCA_023757995.1
A. hordeiaustralica BMP 2776^T	36.96	50.0	1053.85	Illumina HiSeq	11,145	GCA_023758085.1
A. ethzedia BMP 0044^T	36.02	50.5	863.78	Illumina HiSeq	11,096	GCA_023757985.1
A. postmessia BMP 2775^T	34.41	51.0	300.39	Illumina HiSeq	11,767	GCA_024291825.1
A. alternata ATCC 66981^T	33.00	51.5	398.06	Illumina MiSeq	—	GCA_047716545.1
A. arborescens EGS 39-128^T	33.89	51.0	310.87	Illumina GA II	—	GCA_000256225.1
A. rosae BMP 2777^T	32.85	52.0	938.13	Illumina HiSeq	11,336	GCA_024043135.1
A. triticina CBS 763.84^T	33.25	52.5	512.98	Illumina NovaSeq	—	GCA_022609215.1
L6	38.70	50.8	1029.67	Illumina HiSeq	12,781	JBRANC000000000.

Note：“－” indicated unknown.

Table S2. Genome assembly integrity assessment for strain L6.

Property	Number	Percentage (%)
Complete BUSCO	6608	99.6
Missing BUSCOs	25	0.4
Fragmented BUSCOs	4	0.1
Complete and duplicated BUSCOs	4	0.1
Total BUSCO group searched	6641	100

 https://doi.org/10.3390/jof11100710

Table S3. NR, KOG, KEGG, Swiss-Prot, and GO databases in strain L6.

Type	Number
NR	12,463
Swiss-Prot	7666
Pfam	8382
KOG	7713
GO	7555

Table S4. CAZyme profiles of strain L6 and other typical Alternaria strains deposited in CAZyme database.

Strain	GH	AA	GT	CE	CBM	PL	Total
A. postmessia BMP 2775^T	288	164	112	53	37	25	679
A. burnsii CBS107.38^T	286	166	108	53	34	26	673
A. arbusti BMP 1465^T	271	158	105	56	32	21	643
A. triticimaculans BMP 0046^T	259	151	99	52	32	21	614
A. rosae BMP 2777^T	256	153	97	53	32	21	612
A. metachromatica BMP 0045^T	261	147	100	51	31	21	611
A. novae-zelandiae BMP 2774^T	263	143	98	51	34	21	610
A. viburni BMP 2772^T	258	147	99	54	31	21	610
A. incomplexa BMP 0042^T	262	148	98	50	30	21	609
A. hordeiaustralica BMP 2776^T	259	149	95	53	29	20	605
A. ethzedia BMP 0044^T	260	146	95	52	30	21	604
A. infectoria BMP 0036^T	257	142	98	54	31	21	603
L6	267	151	86	56	15	21	596
A. ventricosa BMP 2768^T	262	145	82	53	16	21	579
A. postmessia BMP 2775^T	288	164	112	53	37	25	679
A. burnsii CBS107.38^T	286	166	108	53	34	26	673
A. arbusti BMP 1465^T	271	158	105	56	32	21	643

Table S5. Secondary metabolite BGCs of strain L6 by antiSMASH analysis.

Region	Type	Start	End	Similarity Confidence	Most similar known cluster
Region 1.1	T1PKS	638,856	704,651	High	alternariol
Region 1.2	T1PKS	1,400,046	1,466,528	Low	scytalone/T3HN
Region 1.3	NRP-metallophore, NRPS	2,825,285	2,910,363		
Region 1.4	T1PKS	4,985,797	5,053,622	High	alternapyrone
Region 2.1	terpene	1,314,718	1,347,030		
Region 2.2	NRPS-like	1,844,642	1,907,679		
Region 3.1	NRPS	984,917	1,066,956		
Region 3.2	NRPS-like	1,822,670	1,886,022		
Region 4.1	NRPS-like	1,436,405	1,500,253	High	choline
Region 5.1	terpene	326,132	357,749	Low	squalestatin S1
Region 6.1	terpene	1,075,313	1,107,382		
Region 7.1	T1PKS	552,256	620,175	Low	abscisic acid
Region 9.1	T1PKS	704,452	771,338		
Region 10.1	terpene	180,120	210,719		

Region 10.2	transAT-PKS-like	833,698	950,572	Low	bacillaene
Region 10.3	NRPS	992,923	1,082,715	Low	fengycin
Region 11.1	T1PKS, NRPS	249,303	352,973		
Region 13.1	terpene-precursor	979,752	1,004,538		
Region 14.1	NRPS	64,249	129,787		
Region 14.2	other	622,439	683,857		
Region 15.1	terpene	99,357	134,668		
Region 15.2	terpene	336,235	367,802		
Region 15.3	terpene	423,541	454,562		
Region 15.4	NRPS,T1PKS	908,610	953,166	Low	equisetin
Region 16.1	NRPS	2518	68,447	Low	metachelin C/metachelin A/metachelin A-CE/metachelin B/dimerumic acid 11-mannoside/dimerumic acid
Region 16.2	terpene	460,728	493,042		
Region 17.1	terpene-precursor	312,975	344,076		
Region 18.1	NAPAA	129,461	178,753		
Region 18.2	NRPS-like	439,586	503,341		
Region 22.1	terpene-precursor	480,159	512,827		
Region 24.1	NRPS-like	252,970	316,214		
Region 25.1	NRPS, T1PKS	155,307	227,564		
Region 25.2	terpene	347,382	379,279		
Region 27.1	NRPS	193,179	278,085	Low	surfactin
Region 28.1	fungal-RiPP	99,901	152,651		
Region 29.1	terpene	98,713	130,495	High	terpestacin/preterpestacin 3/preterpestacin 2/preterpestacin 1
Region 29.2	isocyanide-nrp	315,725	384,108		
Region 33.1	T1PKS	1	53,260		
Region 41.1	transAT-PKS-like	1	36,419		
Region 43.1	NRPS	7317	82,802		
Region 46.1	terpene	44,718	77,078	High	clavaric acid
Region 49.1	terpene	21,085	52,450		
Region 58.1	T1PKS	5879	52,926	High	betaenone C/probetaenone I/stemphyloxin II/compound 4/compound 6/compound 7/compound 9/compound 3/dehydroprobetaenone I/compound 5
Region 64.1	transAT-PKS-like	1	23,719		
Region 72.1	NRPS	1	9228		

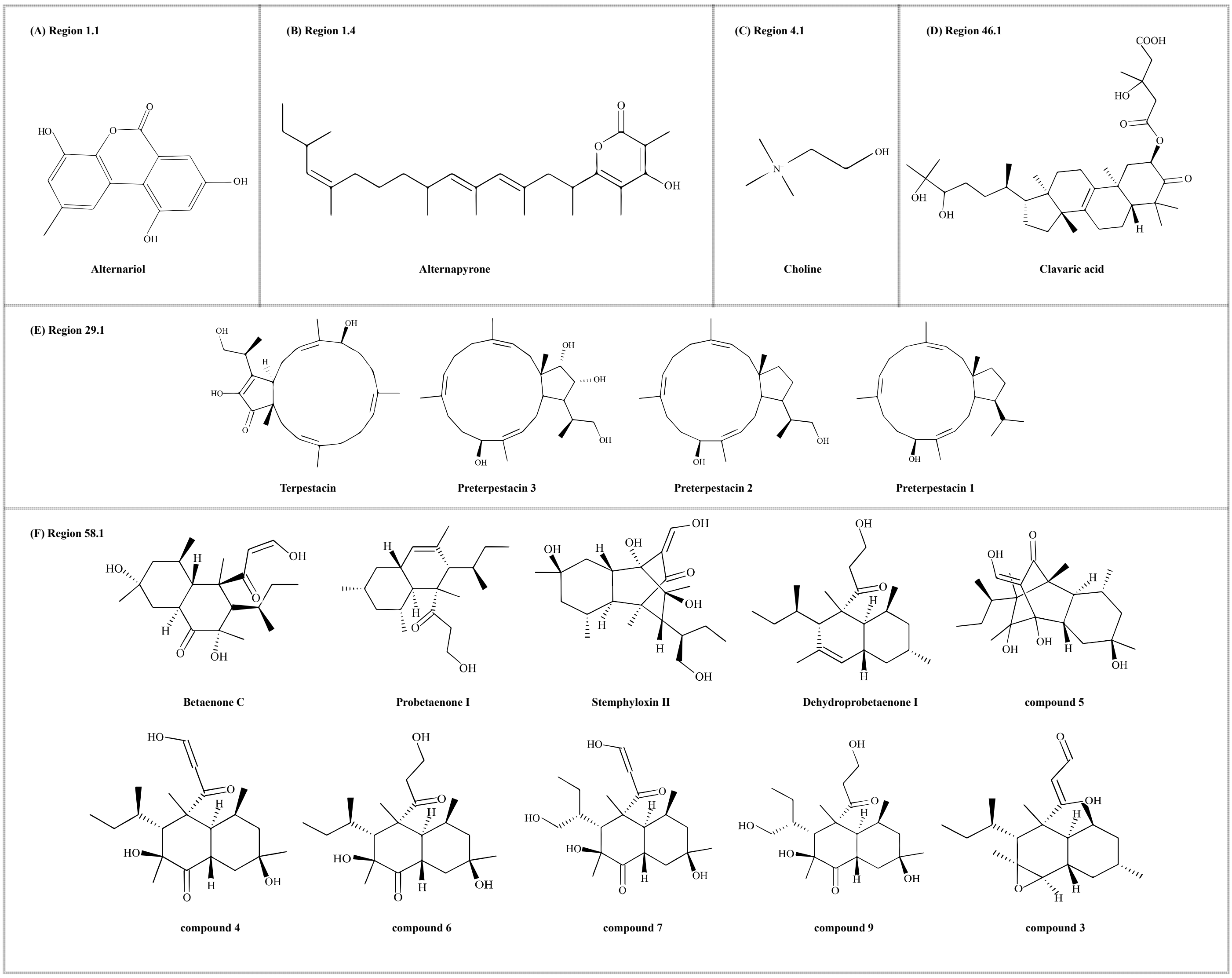

Figure S1. Six identified BGCs with high identity in strain L6 responsible for biosynthesis.

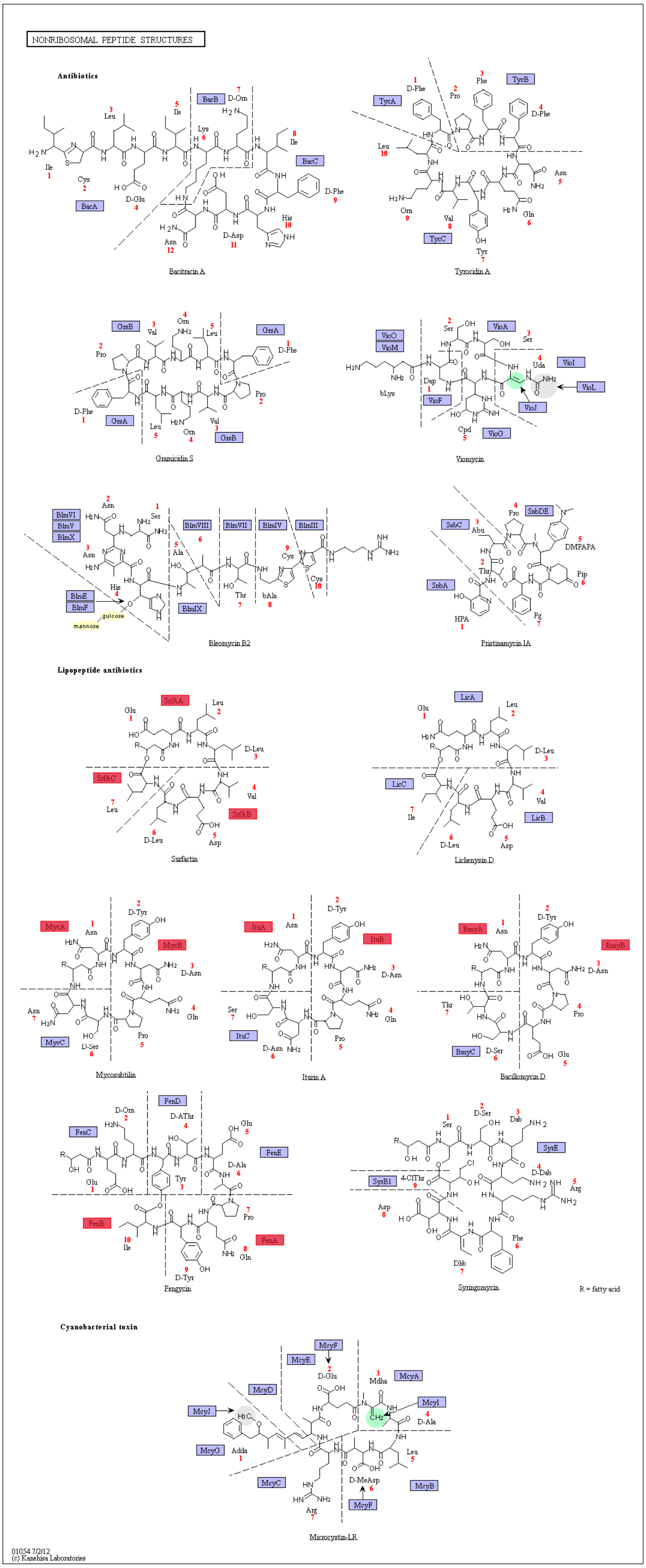

Figure S2. The pathway map for nonribosomal peptide structures (ko01054).
